# Minimally Invasive Isolated Tricuspid Valve Repair After Left-Sided Valve Surgery: A Single-Center Experience

**DOI:** 10.3389/fsurg.2022.837148

**Published:** 2022-03-25

**Authors:** Xiaoyi Dai, Peng Teng, Sihan Miao, Junnan Zheng, Wei Si, Qi Zheng, Ke Qin, Liang Ma

**Affiliations:** ^1^Department of Cardiovascular Surgery, The First Affiliated Hospital, School of Medicine, Zhejiang University, Hangzhou, China; ^2^School of Medicine, Zhejiang University, Hangzhou, China

**Keywords:** tricuspid valve repair, tricuspid valve surgery, tricuspid regurgitation, minimally invasive, redo

## Abstract

**Background:**

Tricuspid regurgitation after left-sided valve surgery was associated with terrible outcomes and high perioperative mortality for surgical treatment. In current years, minimally invasive isolated tricuspid valve repair is increasingly performed in our institution to address tricuspid regurgitation.

**Methods:**

Thirty-seven consecutive patients with previous left-sided valve surgery underwent minimally invasive isolated tricuspid valve repair in our institution between November 2017 and December 2020. Twenty-nine patients(78.4%) were women and the mean age of patients was 58.4 ± 8.5 years. Follow-up was 100% complete with a mean follow-up time of 17.2 ± 9.5 months.

**Results:**

Both the in-hospital and 30-day mortalities were 2.7%. The overall NYHA class had improved significantly during the follow-up (*p* < 0.001). The grade of TR had decreased before discharge (*p* < 0.001) and during the follow-up (*p* < 0.001) compared with the preoperative level although severe TR was recurrent in one patient.

**Conclusions:**

Minimally invasive isolated tricuspid valve repair has acceptable early and midterm outcomes, may be the preferred surgical option to address tricuspid regurgitation after previous left-sided valve surgery when it is feasible.

## Introduction

Tricuspid regurgitation (TR) affects more than 1.6 million people in the USA (1) and is associated with poor clinical outcomes. The prevalence of moderate-to-severe TR is estimated to be as high as 0.55% and up to 3% after 75 years of age; the likelihood of its occurrence is commonly increased in patients with rheumatic heart disease (2, 3), which is prevalent in China. As a result, many patients with TR have undergone left-sided valve surgeries, including mitral valve replacement, aortic valve replacement, or both. When symptomatic TR occurs, patients often choose conservative therapy instead of reoperation due to an inherent fear of undergoing re-median sternotomy and of the high perioperative mortality of isolated tricuspid valve surgery (4, 5).

With the advancement of surgical approaches, minimally invasive access via a right mini-thoracotomy has been implemented in cardiac surgeries, resulting in doubtful patients being more willing to undergo redo surgery. The technical methods involved in tricuspid valve surgery include either tricuspid valve replacement (TVR) or tricuspid valve repair (TVr), but which one is better sremains inconclusive. Although TVR has been widely applied throughout the literature (6–9), some researchers believe that TVr would be the trend in the future (6, 10). Indeed, minimally invasive isolated TVr is increasingly performed in our institution to address secondary TR post-left-sided valve surgery. Therefore, the aim of the present study is to retrospectively evaluate the early and mid-term outcomes of patients who underwent isolated reoperative minimally invasive TVr.

## Methods

### Patients

A total of 37 consecutive patients underwent minimally invasive isolated TVr in our institution between November, 2017 and December, 2020, the specific inclusion criteria are as follows:

1) Who had undergone previous left-sided valve surgery, including concomitant tricuspid valvuloplasty (Table 1).
2) Persistent symptoms after diuretic-based medical treatment due to more than moderate-to-severe TR.
3) Isolated reoperative minimally invasive TVr this time.
4) In absence of severe pulmonary hypertension or significant right ventricle systolic dysfunction.

**Table 1:** Previous left-sided valve surgeries.

**Previous surgery, n (%)**	**Overall (*n* = 37)**
MVR	15 (40.5%)
MVR+AVR	19 (51.4%)
MVR+TVP	1 (2.7%)
MVR+AVR+ TVP	1 (2.7%)
AVR	1 (2.7%)

*AVR, aortic valve replacement; MVR, mitral valve replacement; TVP, tricuspid valvuloplasty*.

Twenty-nine patients (78.4%) were women, and the mean age of the sample was 58.4 ± 8.5 years. The Ethics Committee of the First Affiliated Hospital, College of Medicine, Zhejiang University approved this retrospective study, and informed consent was not required.

### TR Grade Classification

The grade of TR was assessed by transthoracic echocardiography when all patients were admitted. The specific criteria of TR grade were as follows:1+ for mild, 2+ for moderate, 3+ for moderate-to-severe, and 4+ for severe.

### Surgical Procedure

All operations were performed using the standard procedure, which slightly differed according to the operating surgeon's preference. Patients were positioned supine with a 30° elevation of their right side. After heparinization reached the target level, cardiopulmonary bypass (CPB) was established through femoral arterial and venous cannulation. The femoral artery was cannulated with a 16 or 18 Fr arterial perfusion cannula (Edwards Lifesciences, Irvine, California, USA), and the femoral vein was cannulated with a 22 or 24 Fr venous cannula (Edwards Lifesciences, Irvine, California, USA). Subsequently, the position of the femoral venous cannula was adjusted with the use of transesophageal echocardiography until its proximal end reached the inferior vena cava ostium.

A 4–6-cm right anterolateral thoracotomy was performed over the fourth intercostal area, and any potential adhesions were simply separated from the anterior chest wall. When the activated clotting time was >480 s, CPB was started and complete venous drainage was achieved with vacuum assistance. Instead of separating the pericardial adhesions, the right atrium was directly incised along with the pericardium because the included patients had previously undergone cardiac surgery. The balance of arterial inflow and venous drainage was maintained to ensure that the venous return plane in the right atrium was below the tricuspid annulus.

**Figure 1:**
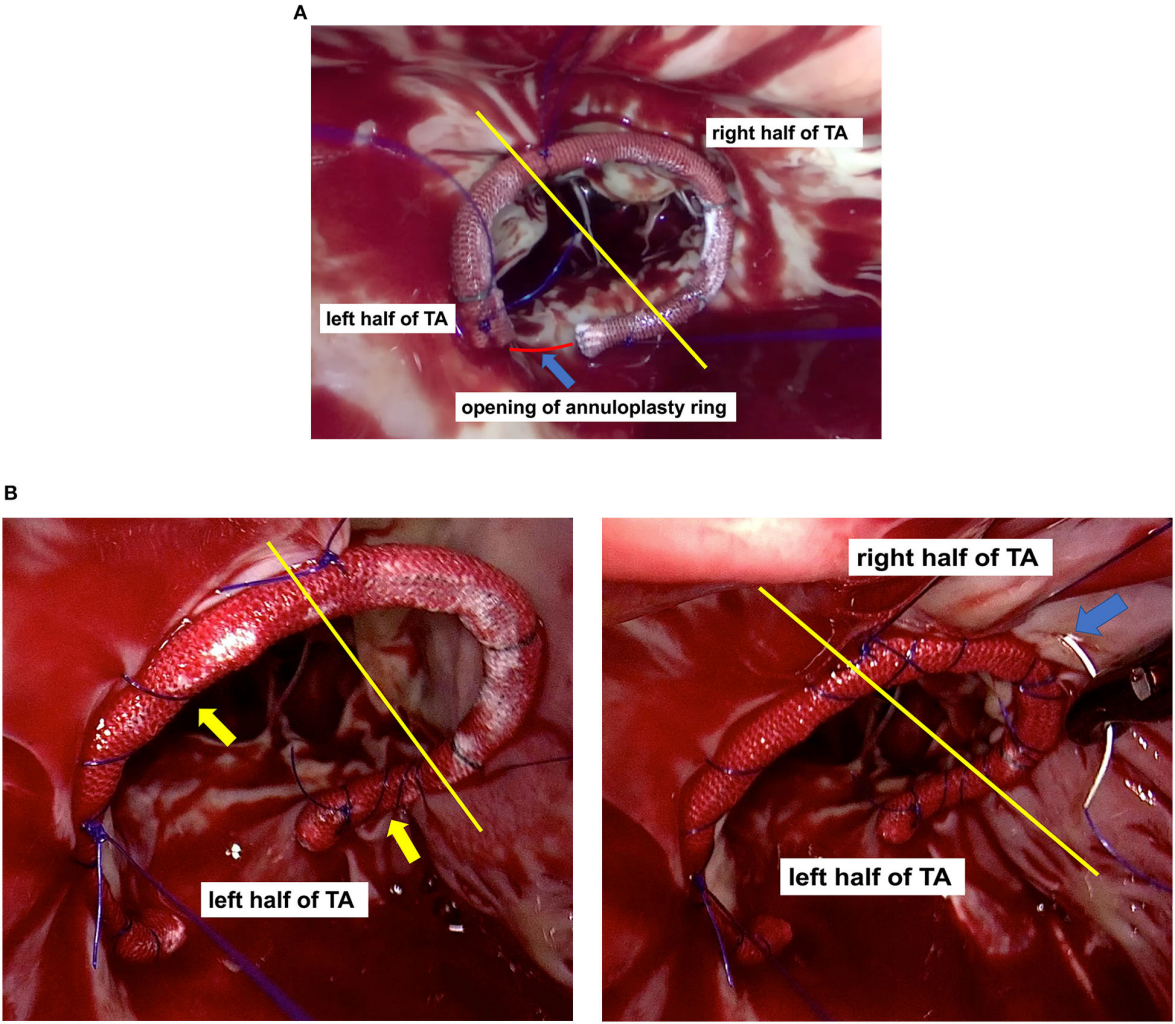
The yellow line divides the tricuspid annulus (TA) into the left and right half parts. **(A)** Annuloplasty ring positioning: Regardless of the mark line on the ring, the opening of the annuloplasty ring (blue arrow) was aligned with the Koch's triangle (red line), and the anterior leaflet segment (or its left part) of the annuloplasty ring was aligned with the anterior leaflets of the tricuspid valve; **(B)** Modified continuous suture: 1) Continuous suturing of the annuloplasty ring to the TA, in a bundling manner (yellow arrow); 2) The right half of the TA (blue arrow) was firmly constricted, whereas its left half was not.

With the assistance of thoracoscopy, all TVr procedures were performed on the beating heart with normothermic CPB. Either a SOVERING™ band (Sorin Group Italia S.r.l, Mirandola, Italy) or an Edwards MC3 annuloplasty ring (Edwards Lifesciences, Irvine, California, USA) was implanted in each patient to correct the annular dilation using a modified continuous suture (Figure 1). The size of the annuloplasty ring was determined by considering the size of the leaflets, length of the annulus, and surface area of the patient's body. The most commonly used size was 28# in female patients and 30# in male patients. Saline was injected into the right ventricle to test valvular competence. If the tricuspid leaflet coaptation was still unsatisfactory, we applied the “edge-to-edge” suture technique to the anterior and septal leaflets (11), or stitched together the midpoint of the tricuspid leaflets' free edges, producing a clover-shaped valve (12).

### Follow-Up

After discharge, all patients or their family members were contacted by telephone and asked about the patients' recent physical condition according to the New York Heart Association (NYHA) functional class. Follow-up was completed by all participants, with a mean time of 17.2 ± 9.5 months (range 3–40 months). Additionally, we asked all patients to undergo periodic echocardiography to assess the TR grade, which was performed either at a local hospital or our institution. The most recent postoperative echocardiographic report was available in 91.7% of the surviving patients (n=36), with a mean follow-up time of 16.5 ± 9.7 months (range 2–40 months).

### Statistical Analysis

Categorical variables were presented as frequency distributions and percentages, and continuous normally distributed variables were expressed as mean ± standard deviation, whereas continuous non-normally distributed variables were expressed as median and first and third quartiles. The Wilcoxon signed-rank test was used to compare the difference between the preoperative and follow-up NYHA classes and TR grades. Statistical significance was set at *p* < 0.05. All statistical analyses were performed using IBM SPSS Statistics (version 26.0; IBM Corp., Armonk, NY, USA).

## Results

The baseline information and clinical characteristics of the included patients are shown in Table 2. The average time elapsed since the previous cardiac surgery was 13.1 ± 5.0 years (range 2–25 years). Almost all patients developed atrial fibrillation, making it the most common comorbidity, and most patients complained of chest tightness/anhelation and leg edema. The high pulmonary artery systolic pressure (PASP) and little tricuspid annular plane systolic excursion (TAPSE) suggested severe degeneration of right ventricular function in this cohort.

**Table 2:** Baseline characteristics.

**Variables**	**Overall (*n* = 37)**
NYHA class III and IV, n (%)	26 (70.3%)
Comorbidities, n (%)	
Permanent pacemaker	4 (10.8%)
Hypertension	8 (21.6%)
Diabetes mellitus	4 (10.8%)
Coronary artery disease	3 (8.1%)
Atrial fibrillation	34 (91.9%)
Anemia	13 (35.1%)
Physical examination, n (%)	
Chest tightness/anhelation	26 (70.3%)
Legs edema	26 (70.3%)
Hepatomegaly	16 (43.2%)
Ascites	4 (10.8%)
Grade of TR, n (%)	
Moderate-severe	8 (21.6%)
Severe	29 (78.4%)
Echocardiography	
LVEF (%)	63.1 ± 7.3
PASP (mmHg)	45.5 ± 9.8
TAPSE (mm)	16 (15–19)
Laboratory data	
TB (μmol/L)	15.9 (10.2–25.0)
BUN (mmol/L)	7.1 (5.9–8.4)
Cre (μmol/L)	68.0 (61.0–86.0)

*BUN, blood urea nitrogen; Cre: creatinine; LVEF, left ventricular ejection fractions; NYHA, New York Heart Association; PASP, pulmonary artery systolic pressure; TAPSE, tricuspid annular plane systolic excursion; TB, total bilirubin*.

Table 3 presents the detailed intraoperative and postoperative outcomes of the patients. Except one patient died postoperatively, in the other 36 patients (97.3%), the procedure was successful. This patient was a 59-year-old woman, with severe stenosis of middle right coronary artery, who developed acute myocardial infarction as soon as the procedure finished. After an urgent percutaneous coronary intervention intraoperatively, she still could not wean off CPB although application of large dose of vasoactive drugs. Finally, the patient died due to liver and kidney failure after 1 day support of extracorporeal membrane oxygenation.

**Table 3:** Surgical details and postoperative outcomes.

**Variables**	**Overall (*n* = 36)**
Operation time (min)	151.4 ± 47.7
CPB time (min)	56 (47-74)
Drainage volume (ml)	250 (120-475)
Prolonged ventilation, n (%)	4 (10.8%)
Duration of ICU stay (d)	3 (2-4)
Duration of hospital stay (d)	8 (7-11)
In-hospital mortality, n (%)	1 (2.7%)
30-day mortality, n (%)	1 (2.7%)

*CPB, cardiopulmonary bypass; ICU, intensive care unit*.

No patient underwent sternotomy, but four patients (10.8%) underwent re-exploration for bleeding, all of which were caused by incomplete hemostasis during surgery. Prolonged ventilation, defined as the requirement for ventilator assistance for more than 72 h, was required in four patients (10.8%). All postoperative complications, including III atrioventricular block, cerebral infarction, and hemodialysis, were only observed in the same patient. Thirty-six patients (97.3%) were discharged and had an uneventful recovery at the subsequent 30-day follow-up.

**Figure 2:**
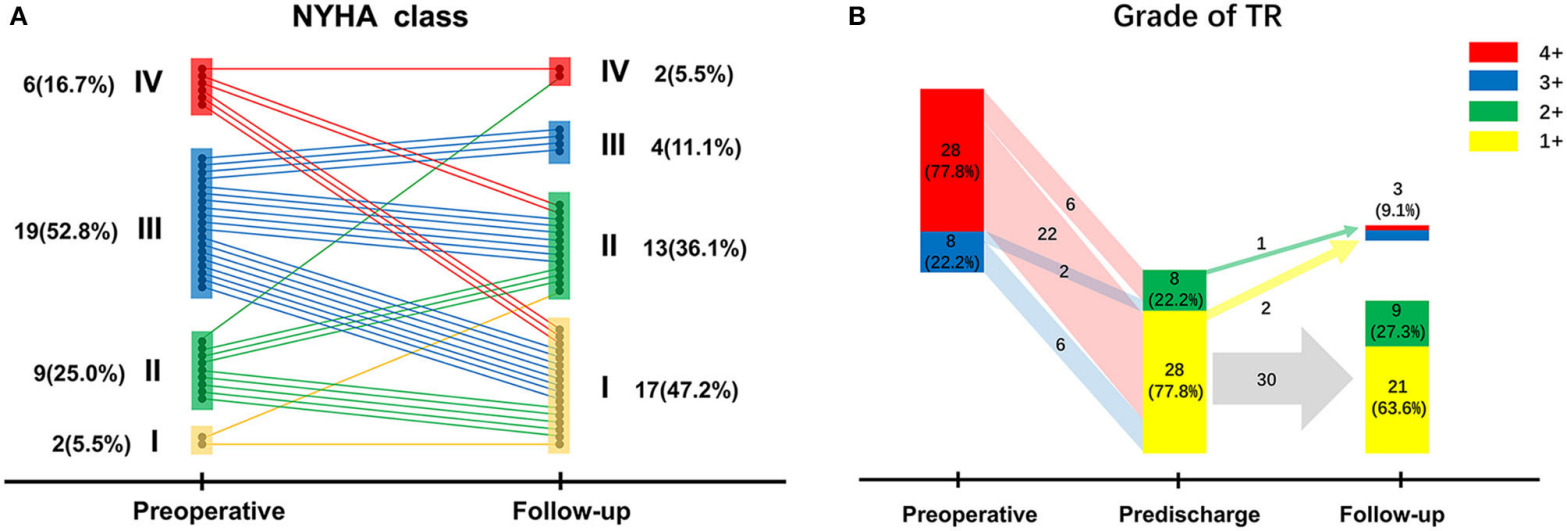
**(A)** New York Heart Association class changes of surviving patients from the preoperative stage to follow-up; **(B)** Tricuspid regurgitation grades of surviving patients preoperatively, before discharge, and at follow-up.

Figure 2A shows the preoperative and follow-up NYHA classes of surviving patients (*n* = 36). Overall, the NYHA class was improved at the follow-up visit compared with that at the preoperative stage (*p* < 0.001); NYHA class improvement was found in 26 patients (72.2%), whereas deterioration from NYHA class II–IV was observed in one patient (2.8%).

The most recent echocardiographic data with a mean follow-up time of 16.5 ± 9.7 months is available in 91.7% of the surviving patients (*n* = 36), which is presented in Figure 2B along with the preoperative and predischarge echocardiographic results. Before hospital discharge, TR grade had decreased significantly compared (*p* < 0.001); indeed, there were no patients with a TR grade ≥3+. However, residual moderate TR was detected in eight patients (22.2%) after TVr; follow-up echocardiographic data were retrieved for this subset of patients, revealing that TR grade decreased in four patients (50.0%) and was stable in three patients (37.5%), but worsened to 4+ in one patient (12.5%). Overall, TR grade had decreased at follow-up (*p* < 0.001) compared with that of the preoperative stage, although a recurrent TR grade ≥3+ was seen in three patients (9.1%), who chose conservative treatment instead of reoperation to address recurrent TR.

## Discussion

Currently, isolated reoperative TVR is more widely applied than TVr across clinical practice, although the latter is regarded as the first choice at our institution. Our experience with this patient series demonstrates that patients undergoing minimally invasive isolated TVr have acceptable early and mid-term outcomes, suggesting that TVr may be the preferred surgical option when feasible.

Appropriate surgical timing is extremely important to consider prior to performing isolated reoperative tricuspid valve surgery. We established that surgical intervention was generally prohibited when TAPSE < 15 mm or PASP > 60 mmHg. Diuretic-based medical treatment was administered to these patients to relieve the volume overload of the right ventricle, and surgery was not to be performed until improvement of the right heart function met surgical indications.

The reported perioperative mortality of isolated tricuspid valve surgeries ranged from 4.2 to 37% (4, 5, 13, 14), while the one of the present study was only 2.7%, which may be credited to the advancement of minimally invasive access, accumulation of surgeons' experience, and improvement of perioperative management. It has been reported that some postoperative outcomes of TVR, including 30-day mortality, rates of permanent pacemaker implantation, and stroke were higher than those of TVr (15). In line with similar research, this study showed that TVr seems to have a slight advantage over TVR regarding the rate of postoperative complications (7). In addition, the CPB time and operation time required for TVr were relatively shorter than those required for TVR, which is potentially explained by the following reasons: 1) Our simplified procedure improved the operative efficiency by avoiding vena cava exposition and snare, as well as the time-consuming superior vena cava cannulation; 2) The time required for annuloplasty ring implantation was shorter than that required for a prosthetic valve. Therefore, the shorter CPB and operation time might lead to a shorter duration of ICU and hospital stay. However, the sample sizes of the aforementioned studies were not large enough to conclude this, and the results should be extrapolated with caution.

In the present study, TVr demonstrated excellent performance in addressing TR and improving heart function. The recurrence rate of severe TR before discharge was zero, which is significantly lower than the previously reported rate of 12.5–14% (5, 14); this higher rate may be largely attributed to inadequate repair techniques and non-optimal annuloplasty rings that were available at that time. Our TVr procedure also showed considerable mid-term outcomes in terms of maintaining tricuspid competency, which may be attributed to our modified continuous suture (16). When dealing with a severely dilated tricuspid annulus, the intermittent mattress suture hardly reduces the tension between the tricuspid annuloplasty ring and the tricuspid fibrous annulus, and potentially causes the horizontal rupture of the fibrous annulus. In contrast, the proposed modified continuous suture was performed on the right atrium wall, creating a larger force-bearing area for the suture and decreasing the stress on the annulus. Additionally, the suture's stitch distance and density can be adjusted, and the range of annulus constriction can also be adjusted after the saline injection test.

The modified continuous suture firmly constricted the right half of the annulus without constricting its left half. The left part of the anterior valve annulus, adjacent to the aortic root, is difficult to constrict due to the presence of aortic pulsation. The left part of the septal valve annulus should not be sutured at great depths because the conduction bundles are located below it. In addition, the left half of the annulus has less suturable tissue; therefore, it results to be weaker in strength and poorer in compliance. Thus, it is not recommended to overly constrict the left half of the annulus. However, since the right half of the annulus does not present any of the aforementioned defects and is not adjacent to risk areas such as the aorta and conduction bundles, it is suitable for constriction.

Although the TR recurrence rate of TVr had been reported to be higher in the short-term (14, 17), whether the bioprosthetic valve with limited durability better maintained long-term tricuspid competency had not been demonstrated. Additionally, after TR correction, NYHA class greatly improved in most patients but did not in approximately one quarter of them, which might be related to the late timing of surgical intervention.

Choosing between TVR and TVr has generated much debate. A previous study reported that there was no significant difference in postoperative NYHA class improvement and right ventricular function maintenance between TVR and TVr (17). Thus, we preferred TVr to TVR due to the high risks of prosthetic thrombosis, excessive anticoagulation, bioprosthetic valve degeneration, and higher rate of postoperative atrioventricular block presented by the latter (15, 18). Moreover, TVr retained all the chordae of the tricuspid valve and preserved the structural integrity of the right ventricle. However, we are still inclined to select TVR as the appropriate treatment for patients with severe leaflet damage, extreme annular dilation, or previously failed TVr.

At present, the selection of mechanical valves vs. bioprosthetic valves in TVR remains controversial. In fact, four mechanical TVRs were performed at our institution before May 2018, when bioprosthetic valve degeneration was considered a challenging clinical issue occurring in relatively young patients, which was attributed to the inadequate percutaneous tricuspid intervention technique used at the time. Pfannmüller (14) held the view that if TVR was inevitable, biological prostheses should be routinely used regardless of age, even in patients with previously implanted mechanical valves, to avoid excessive anticoagulation; this concept was also supported by some studies (6–8) presenting a high application rate of biological prostheses. In fact, the mechanical valve was more prone to thrombosis in the tricuspid position because of the lower pressures and velocities here (19), whereas the bioprosthetic valve was more durable. Additionally, implantation of a mechanical tricuspid valve would make pacemaker placement and right heart catheterization extremely difficult.

## Study Limitations

This study has some significant limitations. First, the sample was small because many symptomatic patients presenting with TR after previous left-sided valve surgery are inclined to undergo conservative treatment instead of isolated tricuspid valve surgery. Another limitation is the short follow-up time; thus, the retrieved echocardiographic data only represent the early effect of TVr, and the rate of freedom from a TR grade ≥3+ cannot be estimated. Therefore, the procedure's long-term effects should be further investigated. Nevertheless, to the best of our knowledge, our cohort represents one of the largest series of minimally invasive isolated TVr via a right mini-thoracotomy reported to date.

## Conclusions

Minimally invasive isolated TVr is a safe and effective procedure to address TR after previous left-sided valve surgery, presenting low perioperative mortality and acceptable early and mid-term outcomes. When TVr is feasible, it will be regarded as our preferred surgical option.

## Data Availability Statement

The original contributions presented in the study are included in the article/supplementary material, further inquiries can be directed to the corresponding authors.

## Ethics Statement

The studies involving human participants were reviewed and approved by First Affiliated Hospital, School of Medicine, Zhejiang University. Written informed consent for participation was not required for this study in accordance with the national legislation and the institutional requirements.

## Author Contributions

XD and PT: conception and design. LM: administrative support. XD, PT, and LM: provision of study materials or patients. XD, SM, WS, QZ, and KQ: collection and assembly of data. XD, PT, and JZ: data analysis and interpretation. All authors writing and final approval of manuscript.

## Conflict of Interest

The authors declare that the research was conducted in the absence of any commercial or financial relationships that could be construed as a potential conflict of interest.

## Publisher's Note

All claims expressed in this article are solely those of the authors and do not necessarily represent those of their affiliated organizations, or those of the publisher, the editors and the reviewers. Any product that may be evaluated in this article, or claim that may be made by its manufacturer, is not guaranteed or endorsed by the publisher.

## Abbreviations

TR: Tricuspid regurgitation
TVR: Tricuspid valve replacement
TVr: Tricuspid valve repair
MVR: Mitral valve replacement
AVR: Aortic valve replacement
TVP: Tricuspid valvuloplasty
CPB: Cardiopulmonary bypass
TA: Tricuspid annulus
NYHA: New York Heart Association
TAPSE: Tricuspid annular plane systolic excursion
PASP: Pulmonary atrial systolic pressure
ICU: Intensive care unit
AVB: Atrioventricular block.
